# Diagnostic Value of Choline PET in the Preoperative Localization of Hyperfunctioning Parathyroid Gland(s): A Comprehensive Overview

**DOI:** 10.3390/biomedicines9030231

**Published:** 2021-02-25

**Authors:** Cristina Ferrari, Giulia Santo, Paolo Mammucci, Antonio Rosario Pisani, Angela Sardaro, Giuseppe Rubini

**Affiliations:** 1 Nuclear Medicine Unit, Interdisciplinary Department of Medicine, University of Bari Aldo Moro, Piazza Giulio Cesare 11, 70124 Bari, Italy; ferrari_cristina@inwind.it (C.F.); giuliasanto92@gmail.com (G.S.); paolo.mammucci@outlook.com (P.M.); apisani71@libero.it (A.R.P.); giuseppe.rubini@uniba.it (G.R.); 2 Section of Radiology and Radiation Oncology, Interdisciplinary Department of Medicine, University of Bari Aldo Moro, Piazza Giulio Cesare 11, 70124 Bari, Italy

**Keywords:** hyperparathyroidism, [^99m^Tc]Tc-MIBI, [^18^F]FCH, choline, positron emission tomography, PET/CT

## Abstract

Hyperparathyroidism is a metabolic disorder characterized by the excessive production of the parathyroid hormone. The diagnosis is based on clinical and laboratory data. In most cases the only treatment is surgery and a correct preoperatory localization of the hyperfunctioning parathyroid gland(s) is essential. Currently, ultrasonography combined with [^99m^Tc]Tc-MIBI parathyroid scintigraphy, optionally associated with single photon emission computed tomography/computed tomography (SPECT/CT), represent the standard preoperative imaging. In recent years, a number of studies have evaluated the potential role of choline positron emission tomography (PET) in hyperparathyroidism with promising results. Most of the recent evidence underlined its higher sensitivity and diagnostic accuracy in the localization of hyperfunctioning parathyroid glands. Choline PET has a higher spatial resolution that is useful for the detection of smaller parathyroid glands and it also has shorter examination times and favorable radiation exposure. These are just a few of the aspects that support it to overcome traditional imaging. Moreover, from the preliminary data, the choline uptake mechanism seems to also have an impact on its better performance. For these reasons, if first used as second level imaging in patients with negative or inconclusive traditional imaging results, several authors have supported its use as a first line investigation. This comprehensive overview aims to provide an accurate description of the preliminary results available in the literature about the use of choline PET/CT in hyperparathyroidism and to compare these results with the performance of traditional imaging methods.

## 1. Introduction

Hyperparathyroidism (HPT) is the third most common endocrine disorder and is characterized by elevated or inappropriately normal parathyroid hormone (PTH) secretion, which regulates calcium and phosphorus metabolism [1].

This disorder is classified into three subgroups according to the pathogenesis. Primary HPT is the most common form, caused by a solitary parathyroid adenoma (80%), followed by four gland hyperplasia (10–15%), multiple adenomas (5%) and parathyroid cancer (<1%) [2]. HPT can also be due to an alteration of calcium homeostasis, frequently associated with a chronic disease that induces the overproduction of PTH. This condition, known as secondary HPT, can evolve into a tertiary form where one or more parathyroid glands begin to produce PTH independently.

In most cases, surgery is the only curative treatment and the correct presurgical detection of the hyperfunctioning parathyroid gland(s) represents a crucial step for patient management.

The most commonly presurgical imaging assessment used is a combination of neck ultrasonography (US) and parathyroid scintigraphy with [^99m^Tc]Tc-MIBI [3,4]. Different scintigraphic protocols can be used such as the single-tracer ([^99m^Tc]Tc-MIBI), the dual-phase/dual-tracer subtraction protocol ([^99m^Tc]Tc-MIBI + [^99m^Tc]TcO^4−^ (pertechnetate) or [^99m^Tc]Tc-MIBI + [^123^I]Iodine) optionally integrated with single photon emission computed tomography (SPECT) or SPECT/computed tomography (CT) acquisition [5,6,7].

However, both of these methods have a few limitations. Low PTH serum levels and ionized calcium, a small adenoma, multiglandular disease and/or concomitant thyroid disease represent some factors associated with a lower scintigraphy performance [8]. Similarly, neck US is highly operator dependent and, in a few cases, is not able to differentiate parathyroid adenomas from reactive cervical lymph nodes as well as to detect ectopic parathyroid glands located in the upper mediastinum [5,7].

Recently, based on occasional findings, the role of positron emission tomography/CT (PET/CT) using choline-labeled radiopharmaceuticals has been highlighted **[9]**.

The rationale of the use of choline-labeled radiopharmaceuticals in this setting of patients could be explained by the different mechanism of the uptake of parathyroid cells compared with [^99m^Tc]Tc-MIBI.

Of note, the parathyroid gland has two major types of cells, chief and oxyphilic cells. Chief cells are small with a big nucleus and poor cytoplasm and are responsible for PTH secretion in which also choline-kinase, a phospholipid/ Ca^2±^ dependent enzyme, seems to play a role. Oxyphilic cells are characterized by an abundant typically acidophilic cytoplasm due to large amounts of mitochondria [10].

While it is well established that [^99m^Tc]Tc-MIBI, a lipophilic cation, concentrates in the mitochondria of parathyroid cells through the transmembrane electrical potential, the exact biological process underlying the choline uptake in HPT is not yet clearly understood.

Similar to [^99m^Tc]Tc-MIBI, thanks to its positive electric charge, choline-labeled radiopharmaceuticals enter through a membrane transporter and accumulate in the mitochondria of both oxyphilic and chief cells. Furthermore, in the chief cells, choline is also phosphorylated by choline-kinase, which is overexpressed in patients with HPT, and used as a component of cell membranes [11,12,13,14].

Therefore, if [^99m^Tc]Tc-MIBI metabolism depends only on the mitochondria uptake, choline-labeled radiopharmaceuticals can exploit a double uptake mechanism that could represent the advantage of choline-PET to [^99m^Tc]Tc-MIBI scintigraphy [15].

**Figure 1:**
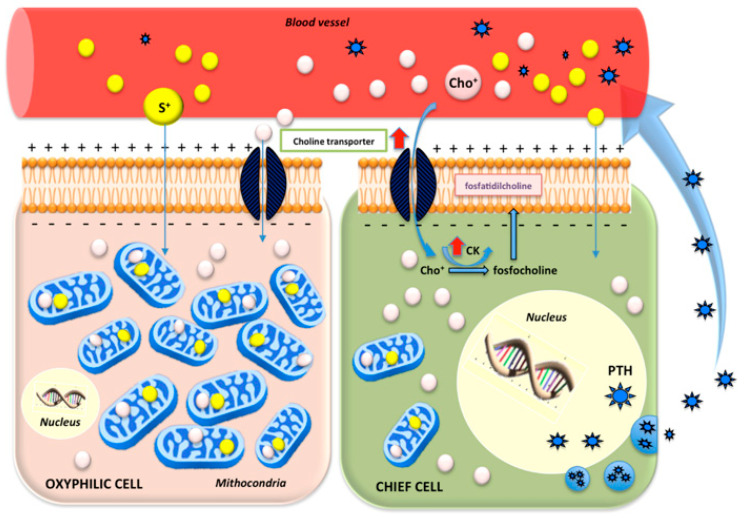
Schematic representation of the dual choline uptake mechanism by chief and oxyphilic cells. Thanks to its positive electric charge, choline enters the parathyroid cells through a membrane transporter, accumulating in both the oxyphilic and chief cells. Furthermore, internalized by chief cells thanks to a transmembrane protein, choline is phosphorylated through choline-kinase and then converted to phosphatidylcholine (lecithin), a necessary component of the cell membrane. The proposal mechanism in hyperparathyroidism (HPT) patients could be explained by an increased intracellular transport and an up-regulation of choline-kinase. The cation [^99m^Tc]Tc-MIBI enters in both parathyroid cells and accumulates in the mitochondria. S^+^: [99mTc]Tc-MIBI; Cho^+^: choline; CK: choline-kinase; PTH: parathyroid hormone.

The different mechanism uptake of both radiotracers in hyperfunctioning parathyroid cells is represented in Figure 1.

The aim of this review is to provide an accurate description of the preliminary results available in the literature about the use of choline PET/CT in HPT and to compare these results with the performance of traditional imaging methods.

## 2. Materials and Methods

A bibliographic literature search until 30 November 2020 was conducted on three electronic databases (Pubmed, Scopus, Google Scholar). Based on a PICO (Problem/Population, Intervention, Comparison, Outcome) strategy search the following keywords were entered: (Hyperparathyroidism AND hyperfunctioning parathyroid glands AND/OR adenoma) and ([^18^F]FCH PET/CT OR [^18^F]FCH PET OR [^11^C]Choline PET/CT AND choline positron emission tomography) and ([^99m^Tc]Tc-MIBI AND parathyroid scintigraphy) and (diagnostic performance AND detection rate AND sensitivity AND specificity). Only original articles edited in English and about humans only that analyzed the role of [^11^C/^18^F]choline-labeled radiopharmaceuticals in the localization of hyperfunctioning parathyroid gland(s) were finally included in this review. Articles with a sample size < 10 patients, review, meta-analysis, case report or case series were not included in the discussion. To identify supplementary eligible articles, additional references were searched from the retrieved review articles.

## 3. Literature Results

The literature search revealed 62 articles. Thirty of them were excluded after reviewing titles, abstracts and full texts. Therefore, 32 studies were considered suitable for the analysis: 14 studies were retrospective whereas 18 studies were prospective. In 28 studies choline was labeled with Fluorine-18 [^18^F] while only four studies evaluated HPT with Carbon-11[^11^C] choline. Most authors (84%) examined the role of choline in primary HPT, in particular in the detection of parathyroid adenoma. Only 16% of authors included patients with secondary and/or tertiary HPT. In 25 studies choline PET/CT was compared with [^99m^Tc]Tc-MIBI scintigraphy. In the articles where sensitivity, specificity, positive predictive value (PPV), negative predictive value (NPV) and overall accuracy were calculated, post-surgical histological examinations and/or the biochemical resolution of HPT after surgery were considered as the gold standard.

The characteristics of the included studies are detailed in Table 1.

### 3.1. Positron Emission Tomography Procedure and Radiation Exposure

As can be seen in Table 1, there was no standard dose administered. The mean dose was 2.5–3 MBq/kg in most of the studies but even lower doses did not appear to have an impact on performance.

A crucial point in parathyroid imaging is the correct timing of the post-injection acquisition that mainly differs in the studies according to radioisotope decay used (only 20 min half-life for ^11^C compared with 110 min for ^18^F). In 15 studies, the authors chose to perform only a single acquisition whereas a dual-phase protocol was the strategy of choice in 16 studies. In particular, a dynamic acquisition was performed in nine studies, an early static acquisition (set at 5 min in most cases) was taken by 12 authors, while a 60 min post-injection acquisition protocol was the most used timing set by the authors. Rep et al. were the only ones to perform a triple time-point protocol.

PET/CT was acquired in the supine position, extending the field of view (FOV) from the base of the skull to the diaphragm in order to include possible ectopic adenomas. None of the authors specified the preferred position of the arms. However, considering a head-neck district investigation, it is highly recommended to perform the scan with the arms down, hyperextending the neck, to avoid any attenuation by the body and reduction of the area visualization. In addition, Michaud et al. suggested extending the acquisition up to mid thighs in men in order not to miss potential prostate foci [17,21].

Regarding radiation exposure, few results are available but promising. Rep et al. conducted a detailed analysis of [^18^F]FCH PET/CT radiation exposure in comparison with parathyroid dual-tracers’ subtraction planar scintigraphy and dual-phase [^99m^Tc]Tc-MIBI SPET/CT. The organ doses and the effective dose (ED) caused by radiopharmaceutical administration were calculated for each method. The median ED after radiopharmaceutical administration resulted in 7.4 mSv (range 6.6–8.2) for parathyroid dual-tracers’ subtraction planar scintigraphy, 5.4 mSv (range 4.8–6.0) for dual-phase [^99m^Tc]Tc-MIBI and 1.9 mSv (range 1.8–3.0) for [^18^F]FCH PET. Moreover, the addition of the early and delayed CT, as part of the hybrid imaging procedures (SPET/CT vs. PET/CT), increased the ED of 26.4% and 42.2% for SPET/CT and PET/CT, respectively. The overall ED (radiopharmaceutical + early/delayed CT) resulted in lower [^18^F]FCH PET/CT than with [^99m^Tc]Tc-MIBI SPECT/CT (2.8 mSv vs. 6.8 mSv) [26].

### 3.2. Diagnostics Performance

In Table 2 the detailed diagnostic performances of the main studies used for the comparison between nuclear medicine imaging methods are reported.

For a correct evaluation of the choline PET/CT diagnostic performance and a reliable comparison with [^99m^Tc]Tc-MIBI scintigraphy, only studies regarding [^18^F]labeled choline PET/CT where both methods were employed as first level imaging were used for the analysis.

In this setting, [^18^F]FCH PET/CT showed a sensitivity(%) of 96.4 ± 2.3 (median 96, range 93.7–100) and 95.0 ± 4 (median 95.8, range 88–100) on per lesion and per patient analysis, respectively, while the sensitivity of parathyroid scintigraphy resulted in 62.9 ± 21 (median 64, range 17–80.7). This low mean value of [^99m^Tc]Tc-MIBI was probably related to the lowest sensitivity reported in one study (only 17%) [27]. However, even excluding these data, the sensitivity of [^18^F]FCH PET/CT remained far higher (lesion-based: 96.4 ± 2.3, median 96, range 93.7–100; patient-based: 94.4 ± 3.8, median 92.8, range 92–100) than a traditional nuclear medicine technique (70.6 ± 8.6, median 70, range 60.8–80.7).

Regarding PPV (%), [^18^F]FCH PET/CT demonstrated a lesion-based value of 95.5 ± 4.9 (median 96.3, range 90.2–100) and a patient-based value of 96.5 ± 3.6 (median 96.7, range 92.8–100) while [^99m^Tc]Tc-MIBI PPV was 97.9 ± 2.7 (median 98.9, range 94.1–100).

The [^18^F]FCH PET/CT overall accuracy (%) resulted in 96.2 ± 0.8 (median 96.3, range 95.3–97) and 95.7 ± 2.6 (median 96.5, range 92.8–98) on a per lesion and per patient analysis, respectively, while the overall accuracy of parathyroid scintigraphy was 85.3 ± 3.9 (median 86.8, range 79.6–88).

Both imaging methods confirmed their high specificity (%): lesion-based 98.6 ± 1.8 (median 99.3, range 96–100) and patient-based 99.3 ± 0.9 (median 99.7, range 98.2–100) for [^18^F]FCH PET/CT and 98.8 ± 1.7 (median 100, range 96–100) for [^99m^Tc]Tc-MIBI.

The comparison suggested a better NPV (%) of [^18^F]FCH PET/CT both on a per lesion (95.2 ± 3.1; median 95.2) and a per patient (96.2 ± 0.2, median 96.2) analysis compared with [^99m^Tc]Tc-MIBI (80.4 ± 9.1, median 80.5).

The detection rate of [^18^F]FCH PET/CT on 411 patients was 92% (91.9 ± 8, median 93.6, range 71–100).

## 4. Discussion

**Figure 2:**
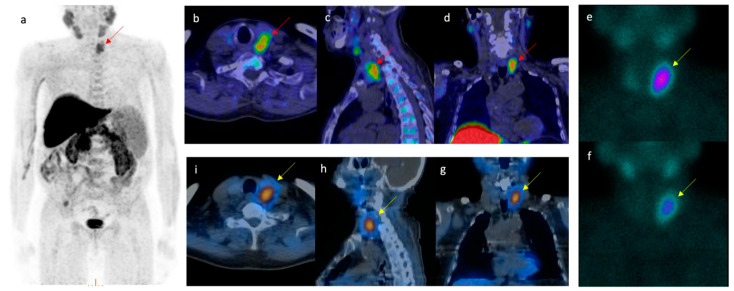
The clinical case of a 72-year-old man who underwent [^18^F]FCH PET/CT (**a**–**d**) revealed a focal [^18^F]FCH uptake posteriorly to the left thyroid lobe (red arrows), of probable parathyroid origin. The patient was directed to laboratory tests that showed a normal level of free triiodothyronine (FT3) (2.94 pg/mL, range 2.20–4.20) and free thyroxine (FT4) (12.4 pg/mL, range 8.10–17.1) as well as a normal thyroid-stimulating hormone (FSH) (1.34 mUI/L, range 0.3–3.6) but elevated PTH (761 pg/mL, range 14–72) and calcium levels (11.1 mg/dL, range 8.5–10.1). Consequently, the patient underwent [^99m^Tc]Tc-MIBI parathyroid scintigraphy (**f**–**i**) according to a dual-phase protocol (**e**,**f**) integrated with single photon emission computed tomography/computed tomography (SPECT/CT) (**g**–**i**). [^99m^Tc]Tc-MIBI identified the same focal area of increased uptake detected by [^18^F]FCH PET/CT (yellow arrows). Following the surgical removal, the pathology examination confirmed the presence of an atypical parathyroid adenoma (Ki67 < 10%) with a post-surgical normalization of PTH and calcium levels. Based on the scientific evidence that proves the superiority of [^18^F]FCH PET/CT compared with [^99m^Tc]Tc-MIBI in hyperparathyroidism, the patient could have avoided other diagnostic investigations, further radiation exposure and spared time for the cure.

We firstly detected occasional findings on choline PET/CT performed for other reasons (i.e., Figure 2). In recent years interest has grown in the use of this multimodality imaging method in the preoperative localization of hyperfunctioning parathyroid glands.

Michaud et al. were one of the first to investigate the role of [^18^F]FCH PET/CT in HPT as second line imaging. In the two studies performed by the same group, a heterogeneous population of patients suffering from both primary and secondary HPT was considered, reaching a lesion-based sensitivity of 89% but a detection rate on a per patient level of 92% [17]. More recently, Uslu-Beşli et al. confirmed the superiority of choline PET when compared with inconclusive traditional imaging methods [41].

Although used as a second line investigation when [^99m^Tc]Tc-MIBI and US imaging were negative or inconclusive, the idea of using choline PET/CT as first line imaging is now supported by promising data.

In most articles of the available literature, choline is [^18^F]labeled, probably due to its favorable characteristics, with only four studies employing [^11^C]labeled choline.

In terms of sensitivity, both [^11^C]labeled and [^18^F]labeled choline showed higher performance compared with traditional imaging methods. The results from a large sample of patients studied by Orevi et al. showed a higher sensitivity of [^11^C]Choline PET/CT (92.3%) when compared with [^99m^Tc]Tc-MIBI (88.5%) in parathyroid adenoma detection [18], up to 98% of sensitivity in other studies [38,42].

Different from [^18^F]labeled choline, the use of [^11^C]labeled choline needs a shorter post-injection acquisition time. Noltes et al. set the optimal tracer uptake time at 20 min post-injection because adenoma choline uptake remains constant from 20 min onwards. Moreover, the same authors suggested a median administered dose of 6.3 MBq/kg and a scanning time for at least 5 min for [^11^C]Choline PET/CT [34].

Regarding [^18^F]labeled choline, the analyzed data demonstrated the high sensitivity and overall accuracy of [^18^F]FCH PET/CT in locating hyperfunctioning parathyroid glands as a result of several factors.

First of all, the higher spatial resolution of PET/CT allows the identification of even smaller lesions [16]. Cuderman et al. showed that lesions with significantly smaller volumes and weights were correctly identified only on PET/CT imaging. The authors found that lesions with a median volume of 4.1cm^3^ and a median weight of 0.5g resulted as a true positive on PET/CT but as a false negative on conventional scintigraphy even if integrated with SPECT/CT [40]. Beheshti et al. confirmed this fact highlighting that the sizes of the parathyroid adenomas were significantly different between negative and positive [^99m^Tc]Tc-MIBI/tetrofosmin SPECT/CT imaging [11].

A noteworthy aspect, directly correlated with a higher spatial resolution, concerns the ability of the [^18^F]FCH PET/CT to detect multiglandular pathology or parathyroid hyperplasia as well as adenomas. In fact, it is known that the diagnostic performance of [^99m^Tc]Tc-MIBI scintigraphy is limited in the case of hyperplasia. The reason is not fully understood but a different mutation in the mitochondrial DNA of the adenoma might be the cause [40,43,44].

Among the collected data, there were no studies that exclusively considered patients with multiglandular pathology or parathyroid hyperplasia. However, in a subanalysis conducted by Cuderman et al., [^18^F]FCH PET/CT showed a sensitivity of 88% in the detection of multiple parathyroid adenoma/hyperplasia compared with 34%, 22%, 22% and 44% of SPECT/CT, subtraction, dual-phase and combined protocols, respectively [40]. In another study, [^18^F]FCH PET/CT reached a sensitivity of 91% compared with 50% of conventional imaging [16].

Although there are no studies that specifically focused on ectopic parathyroid detection, [^18^F]FCH PET/CT has been shown to be superior to conventional imaging. Interestingly, Thanseer et al. reported in their series a case of an ectopic localization in a woman with recurrent HPT who had suffered an accidental implantation of parathyroid tissue in the left breast region (~0.8–0.4 cm) following an endoscopic parathyroidectomy through a periareolar approach five years earlier. Only [^18^F]FCH PET/CT successfully localized the parathyroid adenoma in this rather unusual location [22].

In patients with persistent or recurrent primary HPT after surgery [^18^F]FCH yielded a 95.8% sensitivity when compared with [^99m^Tc]Tc-MIBI (50.0%), US (54.2%) and 4DCeCT (75.0%) [7]. Sensitive preoperative imaging becomes essential for this setting of patients as well as in patients with a history of significant neck surgery such as a total thyroidectomy. In fact, neck surgery often leads to scar formation and/or anatomical modification, increases the risk of recurrent laryngeal nerve injury and is related to post-operative permanent hypoparathyroidism in up to 20% of cases, thus complicating the detection of abnormal parathyroid gland(s) [33,45,46].

Other categories were also investigated. In patients with normocalcemic primary HPT, a milder form with a higher risk of complications [47,48], [^18^F]FCH PET/CT demonstrated a higher detection rate (71%) than US (57%) and [^99m^Tc]Tc-MIBI scintigraphy (6%) [27].

Currently, primary HPT has been the most studied condition because surgical treatment is the standard of care and preoperative localization is essential. For this reason, the literature is poor in studies that exclusively analyzed patients with secondary HPT as well as with tertiary HPT. Only Xue et al. in their prospective study evaluated 17 patients with uremic HPT. The sensitivity, specificity, accuracy, PPV and NPV of [^18^F]FCH PET/CT were 84.13%, 100%, 86.49%, 100% and 52.38%, respectively. In comparison, the corresponding values for [^99m^Tc]Tc-MIBI SPECT/CT were 63.49%, 90.91%, 67.57% and 30.0%. In these patients, [^18^F]FCH PET/CT seemed to be able not only to provide additional information for surgery than traditional imaging methods but also to detect any bone complications that often occur in the tertiary form [36,49].

Finally, two studies compared [^18^F]FCH PET with 4DCeCT, a method that provides both functional and anatomical information thanks to the addition of the fourth dimension consisting of changes in contrast attenuation over time [39]. It was shown that [^18^F]FCH PET/CT and 4DCeCT had quite similar detection rates (57% and 55%, respectively) and sensitivities (81% and 74%, respectively) and, consequently, integrated [^18^F]FCH PET/4DCeCT could yield the highest reliability (73% detection rate and 100% sensitivity) [32].

The results of choline PET in combination with magnetic resonance (MR) imaging are promising (90% sensitivity, 100% PPV in the Kluijfhout et al. study [24]) but more research and a larger sample size are needed [28,35,50].

### 4.1. Pitfalls

The diagnostic performance of choline PET in HPT is affected by both false positive and false negative results.

False positives could be related to malignant disease such as differentiated thyroid carcinoma and/or metastatic lymph nodes as well as other benign conditions including thyroid nodules or inflammatory lymph nodes especially for ectopic locations [7,28].

In the setting of benign lesions, dual time scanning can help with a differential diagnosis; in the late phase, choline uptake decreases both in the thyroid gland parenchyma and inflammatory lymph nodes whereas it increases in parathyroid adenomas [25,26,31]. Similarly, Prabhu et al. reported that early dynamic imaging could also be helpful in differentiating between parathyroid adenomas and cervical reactive lymph nodes that might be confused with small adenomas/hyperplasic glands. The authors demonstrated that the adenoma/thyroid ratio was significantly higher than the lymph node/thyroid ratio (*p* = 0.0117) [30]. Not least, a high uptake of choline has been documented in oncocytic thyroid adenomas [51,52,53].

On the other hand, false negative findings could be related to very small parathyroid adenomas, particularly in a close vicinity to the thyroid gland [32], to the ectopic localization of the parathyroid gland, to multiglandular disease [7] or in cases of intrathyroidal atypical parathyroid adenoma, which is a rare entity with borderline pathological characteristics between adenoma and carcinoma [13,54].

In addition, in patients with autoimmune thyroid disease such as Hashimoto thyroiditis or Graves’ disease a diffuse choline uptake in the thyroid gland can mask a possible parathyroid adenoma resulting in a false negative finding [3,55,56,57]. However, another study documented that the presence of autoimmune thyroid disease did not compromise the use of choline PET/CT from an accurate localization of parathyroid adenoma [15,58].

All of these pitfalls could complicate the correct localization of enlarged parathyroid glands either by US or [^99m^Tc]Tc-MIBI scintigraphy [29,58,59]. However, thanks to the above-mentioned advantages of choline PET/CT in terms of a higher spatial resolution, a better lesion-to-background ratio as well as the added value of semi-quantitative analysis, it overcomes traditional imaging in facing a few of these important issues.

### 4.2. Semi-Quantitative Analysis and Biochemical Data Correlation

As mentioned above, different protocols were studied with special reference to the semi-quantitative parameters.

Prabhu et al. supported the use of dynamic acquisition instead of delayed static imaging, demonstrating that both the maximum standardized uptake value (SUVmax) and the parathyroid/thyroid ratio (P/T ratio) were comparable in the two acquisition time protocols [30].

When comparing early and delayed static acquisitions, several authors observed a better lesion-to-background and lesion-to-thyroid contrast on late images but all lesions were visible at both phases [16,17,21,31].

Other studies found an increase in the SUVmax of the lesion on late images, suggesting that a single late acquisition may be sufficient in patients with high PTH levels [22,23,27].

In the only study considering a triple time-point protocol, Rep et al. found that the optimal scan time was at 60 min after the tracer injection and had a higher accuracy than early images and the same accuracy of images obtained at 120 min (diagnostic accuracies of 94.1%, 96.5% and 96.5% at 5, 60 and 120 min after injection, respectively) [19].

Considering that some lesions could be missed in one-phase acquisition, the dual time-point protocol seemed to be the optimal parathyroid study [31]. More studies are necessary to establish the role of dynamic/early static acquisition not only for comparison with the late images but also for the possibility of performing it as a single scan, reducing examination times especially for uncooperative patients.

Data related to semi-quantitative parameters of choline PET/CT are not uniform and a standardized cut-off does not exist to differentiate towards physiological and pathological parathyroid uptake. In fact, the SUVmax was similar in the case of true positive and false positive uptakes (mean value 4.5 vs. 3.1, respectively) [7]. In a few articles, parathyroid adenoma showed a higher SUVmax than hyperplasic glands (mean value 6.80 vs. 4.53) but the difference was not significant [11]. However, additional information could be obtained by comparing the parathyroid uptake with the background and/or the thyroid uptake [29]. The P/T ratio ranged from 1.2 to 2.5 on average [11,20,21].

A few authors investigated whether the SUVmax may be predictive of disease severity by exploring a possible correlation between the SUVmax values of hyperfunctioning parathyroid gland(s) and serum PTH, Ca, phosphorus (P) levels and bone mineral densitometry (BMD) results. In 2018, a retrospective study of 52 patients highlighted a strong positive correlation between the SUVmax and the PTH level as well as between the adenoma-to-background ratio and the PTH level on both late and early phases [25].

Morland et al. identified a significant association between [^18^F]FCH PET/CT and higher calcium levels; in their study, [^18^F]FCH PET/CT was performed during Cinacalcet treatment in 23% of patients [32]. Cinacalcet is a modulator of the calcium-sensing receptor strongly expressed on the surface of parathyroid cells that reduces serum calcium and PTH levels in patients for whom surgery is not clinically indicated or contraindicated. The authors found that Cinacalcet did not negatively impact the [^18^F]FCH PET/CT results. Rather, patients displaying positive [^18^F]FCH PET/CT tended to be more frequently under Cinacalcet treatment presumably because of higher calcium levels [1,60,61]. Interestingly, calcium was the only parameter that showed a significant association with positive findings on integrated [^18^F]FCH PET/4DCeCT [32].

The correlation between the SUVmax and densitometric results was also examined. Serum PTH levels were significantly higher and femoral/lumbar T values were lower in patients with an SUVmax greater than 4.4 [13]. Choline uptake seemed to be correlated with the molecular profile too; a significant correlation between the SUVratio and Ki67 expression and an inverse correlation between SUV parameters (SUVmax; SUVratio) and p53 expression was demonstrated [32].

At the time of writing this review, the phase III clinical trial APACH2 is ongoing [62]. As highlighted in this literature overview, the trial aims to demonstrate that the high diagnostic performance of [^18^F]FCH PET/CT could improve the outcome of patients, resulting in a saving in terms of total clinical care costs. Preliminary results are waited for to confirm the direction in the near future.

## 5. Conclusions

In conclusion, due to the high diagnostic value demonstrated by the reported data, choline PET could represent the ideal method to replace traditional imaging in the preoperative localization of hyperfunctioning parathyroid gland(s), demonstrating it to be a significantly more sensitive method in different patient settings than any conventional scintigraphic approach used.

## Figures and Tables

**Table 1:** Characteristics of the original articles reviewed.

Year	Isotope	Authors’ PMID	Study Type	Pts	Dose (MBq)	Time p.i. (min)	Field of View (FOV)	Scintigraphic Protocol	Performance	Main Findings
**2014**	^18^F	Lezaic et al. [16] 25063039	P	24	100	5 60	Neck and upper mediastinum	ALL	Sensitivity, specificity, PPV, NPV	PTA: [^18^F]FCH PET/CT sensitivity 94%; specificity 100%. Multiple parathyroid lesions: [^18^F]FCH PET/CT sensitivity 91% (vs. conventional imaging, 50%).
**2014**	^18^F	Michaud et al. [17] 26469908	P	12	3/kg	Dynamic	From the skull to mid thighs (men) or to liver (women)	Dual-tracer/dual-phase SPECT/CT	Sensitivity, DR	On a per lesion level, [^18^F]FCH PET/CT sensitivity was 89%. On a per patient level, the DR of [^18^F]FCH PET/CT was 92%.
**2014**	^11^C	Orevi et al. [18] 25290292	P	40	370	Dynamic	Middle ear to diaphragm	Dual-tracer/dual-phase SPECT/CT	SUVmax SUVratio sensitivity	Lesions SUVmax ranged from 2.1 up to 31. The SUVratio ranged from 0.85 to 4.31. [^11^C]Choline yielded a sensitivity of 92.3% compared with 88.5% of [^99m^Tc]Tc-MIBI.
**2015**	^18^F	Rep et al. [19] 26834518	P	43	100	5 60 120	Neck and upper mediastinum	\	Sensitivity, specificity, PPV, NPV, accuracy, SUVmax, SUVratio	Accumulation of [^18^F]FCH PET/CT was higher in parathyroid lesions in comparison with the thyroid tissue with a significantly higher SUVmean in the second (60 min) and in the third (120 min) phase, with a better contrast lesions/thyroid tissue in the second and the third phase.
**2016**	^18^F	Kluijfhout et al. [20] 27612033	R	44	2/kg	30	From the base of the skull to the mediastinum	Dual-tracer/dual-phase SPECT/CT	DR, sensitivity, PPV, SUVratio	[^18^F]FCH PET/CT was positive in 34/44 (77.3%) cases. In patients who underwent surgery (33), [^18^F]FCH PET/CT showed a sensitivity 94.3% (vs. 30% of [^99m^Tc]Tc-MIBI) and a PPV of 97.1% (vs. 69.2%). The mean SUVmax of the 33 preoperatively identified pathological glands was 5.2, which was significantly higher than the 2.9 SUV of the thyroid.
**2016**	^18^F	Michaud et al. [21] 26469908	P	17	3/kg	Dynamic 10	From the skull to mid thighs (men) or to liver (women)	Dual-tracer/dual-phase SPECT/CT	Sensitivity specificity SUVmax SUVratio	Per patient-based, the scintigraphy sensitivity was 69% by open and 94% by masked reading and [^18^F]FCH PET/CT sensitivity was 88% by open and 94% by masked reading. Per lesion-based, the scintigraphy sensitivity was 58% by open and 83% by masked reading and [^18^F]FCH PET/CT sensitivity 88% by open and 96% by masked reading.
**2017**	^18^F	Thanseer et al. [22] 28902729	P	54	150–185	10–15; 60	\	Dual-phase SPECT/CT	Sensitivity, PPV, accuracy	[^18^F]FCH PET/CT showed 100% sensitivity, 96.3% PPV and 96.3% accuracy in localizing parathyroid adenomas regardless of the location (eutopic or ectopic) on patient-wise analysis. In the lesion-wise analysis of 56 lesions, [^18^F]FCH PET/CT revealed 100% sensitivity, 92.8% PPV and 92.8% accuracy.
**2017**	^18^F	Hocevar et al. [23] 27776943	R	15	100	5; 60	Neck and upper mediastinum	Dual-phase SPECT/CT	PPV	[^18^F]FCH PET/CT showed a PPV of 95.2% according to the histology.
**2017**	^18^F	Kluijfhout et al. [24] 28121522	P	10	188 ± 26	Dynamic 10	From the base of the skull to the mediastinum	Dual-tracer/dual-phase SPECT/CT	Sensitivity, PPV, SUVratio	[^18^F]FCH PET/MR reached 90% sensitivity without any false positive results (PPV: 100%). The median SUVmax was 4.9, significantly higher than the 2.7 of thyroid SUVmax (SUVratio ranging from 1.2 to 2.5).
**2018**	^18^F	Alharbi et al. [25] 29508264	R	52	150 ± 12	2; 50	Top of the head to the upper abdomen	\	SUVmax	The majority of adenomas (71.1%) demonstrated an increase in SUVmax from early to late phase. A positive correlation was observed between SUVmax in late phase and PTH level and between ABR in late phase and PTH level. The surgical specimen volume was positively correlated with the PET/MR volume and PTH level.
**2018**	^18^F	Rep et al. [26] 29339573	P	36	100	5; 60	Neck and upper mediastinum	Dual-phase SPECT/CT	Sensitivity, specificity, ED	PSS: sensitivity 46% and specificity 98%. [^99m^Tc]Tc-MIBI SPECT/CT sensitivity 64% and specificity 96%. [^18^F]FCH PET/CT sensitivity 97% and specificity 99%. The ED after administration of the RPH alone was highest for conventional dual-tracer imaging followed by [^99m^Tc]Tc-MIBI alone (SPECT/CT); it was the lowest for [^18^F]FCH (PET/CT).
**2018**	^18^F	Araz et al. [13] 30138157	P	35	100	45–60	\	Dual-phase SPECT/CT	Sensitivity, specificity, PPV, NPV, accuracy, SUVmax	The sensitivity, specificity, PPV, NPV and accuracy were 96, 100, 100, 93 and 97%, for [^18^F]FCH PET/CT, respectively. The mean serum PTH levels were significantly higher and lumbar and femur T scores were significantly lower in patients with an SUVmax greater than 4.4.
**2018**	^18^F	Bossert et al. [27] 30094743	P	34	3–3.5/kg	9; 60	\	Dual-tracer/dual-phase SPECT/CT	DR, sensitivity	In the whole patients group the DR was 68% for US, 71% for [^18^F]FCH PET/CT and 15% using [^99m^Tc]Tc-MIBI. In the subgroup of normocalcemic, [^18^F]FCH PET/CT had a higher DR (71%) compared with traditional imaging. Sensitivities were 88%, 82% and 17% for [^18^F]FCH PET/CT, US and [^99m^Tc]Tc-MIBI, respectively.
**2018**	^18^F	Grimaldi et al. [7] 29680989	P	27	100	30	\	Dual-phase SPECT/CT	Sensitivity, specificity, PPV, NPV.	In patients with HPT recurrence or with a suspicion of multiple gland disease, [^18^F]FCH PET/CT showed a per patient sensitivity of 81%, PPV 94% and a per gland sensitivity of 76%, PPV 85%, specificity 91% and NPV 86%.
**2018**	^18^F	Huber et al. [28] 29372312	R	26	151 ± 16	Dynamic	From the vortex to the upper abdomen	Dual-phase SPECT/CT	Sensitivity, PPV	FCH PET sensitivity was 96.2%, PPV 100%.
**2018**	^18^F	Quak et al. [29] 29270788	P	25	1.5/kg	60	\	Dual-phase SPECT/CT	SUVratio, SUVmax, sensitivity, PPV	On per lesion and per patient [^18^F]FCH PET/CT sensitivities of 91.3% and 90.5%, respectively. On per lesion and per patient PPV were 87.5% and 86.4%, respectively. [^18^F]FCH uptake in adenomas was five and a half-fold higher than surrounding muscle background and three-fold higher than thyroid uptake.
**2018**	^18^F	Prabhu et al. [30] 30379751	P	14	185–296	Dynamic 45–60	From the angle of mandible to the diaphragm	\	A/T ratio; LN/T ratio; SUVmax	A higher SUVmax was observed in dynamic images compared with static images. A/T ratio in the dynamic and static images was comparable. The tracer may be able to adequately differentiate parathyroid adenoma from cervical LN: the difference between mean A/T and LN/T ratios in early dynamic imaging was found to be significant.
**2018**	^18^F	Zajíčková et al. [15] 30484682	R	13	180 ± 48	30 ± 20	From the base of the skull to the mediastinum	Dual-tracer/dual-phase SPECT/CT	Sensitivity, PPV	[^18^F]FCH yielded a per patient sensitivity of 92%. The per patient PPV of [^18^F]FCH was 100%. The mean size of 14 resected parathyroid lesions was 11.9 mm with a per lesion sensitivity of 93% and per gland PPV of 81%.
**2018**	^18^F	Beheshti et al. [11] 29516131	P	82	3.2/kg	60; ± 100–120	From the base of the skull to the mediastinum	Dual-phase SPECT/CT	DR, sensitivity, specificity, PPV, NPV, accuracy	DR: [^18^F]FCH PET/CT 93% vs. [^99m^Tc]Tc-MIBI SPECT/CT 61%. The sensitivity, specificity, PPV, NPV and overall accuracy of PET/CT were 93.7%, 96.0%, 90.2%, 97.4% and 95.3%, respectively. Corresponding values for [^99m^Tc]Tc-MIBI SPECT/CT were 60.8%, 98.5%, 94.1%, 86.3% and 87.7%, respectively. The sizes of the parathyroid adenomas significantly impacted on [^99m^Tc]Tc-MIBI SPECT/CT.
**2019**	^18^F	Broos et al. [31] 30877179	R	64	150	5 60	From the temporomandibular joint to the diaphragm	\	SUVmax; SUVpeak; SUVratio	There was a significant decrease in [^18^F]FCH uptake in the glands on late versus early time-points but there was a significant increase in the ratio of parathyroid uptake to thyroid uptake.
**2019**	^18^F	Piccardo et al. [32] 30219964	P	44	2.5/kg	10	From the upper neck to abdomen	Dual-tracer/dual-phase SPECT/CT	DR, sensitivity	[^18^F]FCH PET/4DCeCT detection rate was 72.7% and in surgically treated patients the sensitivity was 100%. These results were significantly better than those of [^18^F]FCH PET/CT (56.8% and 80%, respectively) and those of 4DCeCT (54.5 and 74%, respectively).
**2019**	^18^F	Amadou et al. [33] 30659347	R	29	231 ± 42	60	\	Dual-phase SPECT/CT	Sensitivity specificity, PPV, NPV	On a per lesion analysis, sensitivity, specificity, PPV and NPV values were, respectively, 96%, 13%, 77% and 50% for [^18^F]FCH PET/CT and 75%, 40%, 80% and 33% for 4DCeCT. On a per patient analysis, sensitivity was 85% for [^18^F]FCH PET/CT and 63% for 4DCeCT.
**2019**	^11^C	Noltes et al. [34] 31367792	R	21	6.3 ± 1.2 /kg	20;	\	\	SUVratio SUVmax	The A/T ratio became constant from the uptake time of 20–30 min p.i. onwards. In the uptake time of 0–10 vs. 10–20 min p.i. the A/T ratio significantly increased from 1.49 to 1.65.
**2019**	^18^F	Khafif et al. [35] 30877424	P	19	100	Dynamic	\	Dual-phase SPECT/CT	Sensitivity	[^18^F]FCH PET/MR showed a sensitivity of 84.2% and predicted the side of the disease gland in 100% of cases.
**2019**	^18^F	Xue et al. [36] 31879808	P	17	111–185	10 60	Neck and upper mediastinum	Dual-tracer/dual-phase SPECT/CT	Sensitivity, specificity, PPV, NPV, accuracy	In patients with uremic hyperparathyroidism [^18^F]FCH PET/CT showed a sensitivity, specificity, accuracy, PPV and NPV of 84.13%, 100%, 86.49%, 100% and 52.38%, respectively.
**2019**	^18^F	Broos et al. [37] 31367807	R	271	150	5 60	From the temporomandibular joint to the diaphragm	\	DR; SUVmax	DR on a per patient level: 96%; DR on a per lesion level 90%. The mean SUVmax on the early images was 6.1 and decreased to a mean SUVmax of 4.9 on the late images.
**2020**	^18^F	Morland et al. [1] 31914064	R	47	2.5/kg	Dynamic 60	\	Dual-tracer/dual-phase SPECT/CT	DR	DR of [^18^F]FCH PET/CT was 62%. [^18^F]FCH PET/CT positivity was associated with a higher calcium level. Cinacalcet treatment did not impact on PET positivity.
**2020**	^11^C	Liu et al. [38] 31601698	R	87	385 ± 175	20	Neck and upper chest	Dual-tracer SPECT/CT	Sensitivity, PPV, SUVmax; SUVratio(carotid); SUVratio(thyroid)	[^11^C]Choline sensitivity was 98.8%. The corresponding lesioned PPV was 91.3%. The mean SUVmax was 6.15 ± 4.92 in 72 lesions with a positive uptake (70 patients).
**2020**	^18^F	Pretet et al. [39] 32604786	R	50	2/kg	60	From the mandible to the carina	Dual-tracer SPECT/CT	Sensitivity, DR	On a per patient analysis, sensitivity was 93%, 80% and 95% and DR% was 82%, 68% and 84%, respectively for PET/CT, 4DCeCT and PET/4DCeCT. On a per gland analysis, sensitivity PET/CT, 4DCeCT and PET/4DCeCT was 88%, 66% and 92% and DR% was 79%, 57% and 83%, respectively. PET/CT and PET/4DCeCT were more sensitive than 4DCeCT alone.
**2020**	^18^F	Cuderman et al. [40] 31562221	P	103	100	5 60	Neck and upper mediastinum	ALL	Sensitivity, specificity	[^18^F]FCH PET/CT demonstrated a sensitivity of 92% superior to all scintigraphic modality methods (39–56%) on a per patient level. A better sensitivity of [^18^F]FCH PET/CT was shown in multiple parathyroid adenoma/hyperplasia detection (88% vs. 44%).
**2020**	^18^F	Uslu-Beşli et al. [41] 31997599	R	105	325.1 ± 86.7	15 45	Neck and upper chest (15 min); whole body (45 min)	Dual-phase SPECT/CT	Sensitivity, PPV, accuracy	Sensitivity, PPV and accuracy of [^18^F]FCH PET/CT in the detection of HPT were 94.1%, 97.9% and 92.4%, respectively. A single time-point imaging could be associated with a potential risk of missing lesions.
**2020**	^11^C	Ismail el al. [42] 33228254	P	60	400	10 ± 5	\	Dual-tracer SPECT/CT	Sensitivity	At the patient level, sensitivities were 0.98 for dual-tracer subtraction scintigraphy and 1.00 for [^11^C]Choline PET/CT. At the gland level, sensitivities were 0.88 and 0.87, respectively.

p.i.: post-injection; P: prospective; R: retrospective; [^18^F]FCH PET/CT: [^18^F]labelled fluorocholine positron emission tomography/computed tomography; US: ultrasonography; PPV: positive predictive value; NPV: negative predictive value; PTA: parathyroid adenoma; DR: detection rate; SUV: standardized uptake value; ABR: adenoma to background ratio; PTH: parathyroid hormone; PSS: planar scintigraphy subtraction; ED: effective dose; RPH: radiopharmaceutical; A/T: adenoma/thyroid; LN/T: lymph node/ thyroid.

**Table 2:** Diagnostic performance of [^18^F]FCH PET/CT vs. parathyroid [^99m^Tc]Tc-MIBI scintigraphy.

			[^18^F]FCH PET/CT *										[^99m^Tc]Tc-MIBI **				
	Author-PMID	HPT	Per Lesion (%)					Per Patient (%)									
			Sen	Spe	PPV	NPV	Acc	Sen	Spe	PPV	NPV	Acc	Sen	Spe	PPV	NPV	Acc
FIRST LEVEL IMAGING	Thanseer et al., 2017 [22]	I	100	\	96.3	\	96.3	100	\	92.8	\	92.8	80.7	\	97.7	\	79.6
	Beheshti et al., 2018 [11]	I	93.7	96.0	90.2	97.4	95.3	\	\	\	\	\	60.8	98.5	94.1	86.3	87.7
	Araz et al., 2018 [13]	I	96	100	100	93	97	\	\	\	\	\	78	100	100	70	86
	Lezaic et al., 2014 [16]	I	\	\	\	\	\	92	100	100	96	98	64	100	100	85	88
	Rep et al., 2018 [26]	I	97	99	\	\	\	\	\	\	\	\	64	96	\	\	\
	Cuderman et al., 2020 [40]	I	95.5	99.7	\	\	\	92	99.7	\	\	\	76	100	\	\	\
	Bossert et al., 2018 [27]	I	88	\	\	\	\	\	\	\	\	\	17	\	\	\	\
	Rep et al., 2015 [19]	I	\	\	\	\	\	93.6	98.2	96.7	96.4	96.5	\	\	\	\	\
SECOND LEVEL IMAGING	Quak et al., 2018 [29]	I	91.3	\	87.5	\	\	90.5	\	86.4	\	\	\	\	\	\	\
	Zajíčková et al., 2018 [15]	I	93	\	81	\	\	92	\	100	\	\	\	\	\	\	\
	Uslu-Beşli et al., 2020 [41]	I	\	\	\	\	\	94.1	\	97.9	\	92.4	45.1	\	97.9	\	45.7
	Michaud et al., 2016 [21]	I; II	96	56	\	\	\	\	\	\	\	\	83	56	\	\	\
	Kluijfhout et al., 2016 [24]	I; III	94.3	\	97.1	\	\	\	\	\	\	\	30	\	69.2	\	\

HPT: hyperparathyroidism; I: primary hyperparathyroidism; II: secondary hyperparathyroidism; III: tertiary hyperparathyroidism; Sen: sensitivity; Spe: specificity; PPV: positive predictive value; NPV: negative predictive value; Acc: accuracy. * values refer to [18F]FCH PET/CT acquisition at 60 min post-injection. ** in cases of different [99mTc]Tc-MIBI protocols employed, the one with the highest performance values was reported.

## Data Availability

Not applicable.
